# The Root of *Atractylodes macrocephala* Koidzumi Prevents Obesity and Glucose Intolerance and Increases Energy Metabolism in Mice

**DOI:** 10.3390/ijms19010278

**Published:** 2018-01-17

**Authors:** Mi Young Song, Soo-Kyoung Lim, Jing-Hua Wang, Hojun Kim

**Affiliations:** 1 Department of Rehabilitation Medicine of Korean Medicine, College of Korean Medicine, Dongguk University, Dongdaero 123, Gyeongju-si 38066, Korea; 2 Department of Rehabilitation Medicine of Korean Medicine, College of Korean Medicine, Dongguk University, Donggukro 27, Ilsandong-gu, Goyang-si 10326, Korea; sklim0214@gmail.com (S.-K.L.); ewccwang@gmail.com (J.-H.W.); kimklar@gmail.com (H.K.)

**Keywords:** Atractylodis Rhizoma Alba, obesity, glucose intolerance, skeletal muscle, brown fat, energy metabolism

## Abstract

Targeting energy expenditure offers a strategy for treating obesity more effectively and safely. In previous studies, we found that the root of *Atractylodes macrocephala* Koidzumi (Atractylodis Rhizoma Alba, ARA) increased energy metabolism in C2C12 cells. Here, we investigated the effects of ARA on obesity and glucose intolerance by examining energy metabolism in skeletal muscle and brown fat in high-fat diet (HFD) induced obese mice. ARA decreased body weight gain, hepatic lipid levels and serum total cholesterol levels, but did not modify food intake. Fasting serum glucose, serum insulin levels and glucose intolerance were all improved in ARA treated mice. Furthermore, ARA increased peroxisome proliferator-activated receptor gamma coactivator 1 alpha (PGC1α) expression, and the phosphorylation of adenosine monophosphate-activated protein kinase (AMPK) in skeletal muscle tissues, and also prevented skeletal muscle atrophy. In addition, the numbers of brown adipocytes and the expressions of PGC1α and uncoupling protein 1 (UCP1) were elevated in the brown adipose tissues of ARA treated mice. Our results show that ARA can prevent diet-induced obesity and glucose intolerance in C5BL/6 mice and suggests that the mechanism responsible is related to the promotion of energy metabolism in skeletal muscle and brown adipose tissues.

## 1. Introduction

Obesity develops when energy intake exceeds energy expenditure. To combat obesity and obesity-induced insulin resistance, increasing energy expenditure is more effective in the long-term than reducing energy intake, such as by appetite suppression [1,2]. Skeletal muscle is the major organ responsible for increasing energy expenditure by oxidative capacities contributing thermogenesis [3], and adaptive thermogenesis also occurs in brown fat [4]. Furthermore, lipids in skeletal muscle have been extensively studied in the context of insulin sensitivity and glucose tolerance [5]. However, few drugs have been discovered that target energy expenditure for the treatment of obesity.

In traditional Korean medicine (TKM) or traditional Chinese medicine (TCM), “qi” deficiency is considered as being associated with a cold constitution, and sluggish metabolism, hence being a cause of metabolic disorders, such as obesity [6,7,8]. From this viewpoint, herbal drugs, like qi invigorating herbs, offer an empirical rationale that could be applied to the treatment of metabolic syndrome [9,10]. However, few studies have investigated the abilities of qi invigorating herbs to treat obesity.

The root of *Atractylodes macrocephala* Koidzumi (Atractylodis Rhizoma Alba, ARA) is a qi invigorating herbal components [11], and in TKM and TCM ARA is used to treat obesity and diabetes [12]. ARA or fermented ARA have been reported to reduce body weights and serum lipid levels in high fat diet (HFD) induced animal models of obesity [13,14], but the mechanisms responsible for these anti-obesity effects have not been elucidated. In a previous study, we found ARA increased lipid and glucose metabolism by enhancing the activities of peroxisome proliferator-activated receptor gamma coactivator 1 alpha (PGC1α) and adenosine monophosphate-activated protein kinase (AMPK) in C2C12 skeletal muscle cells [15], and, in a following study, we showed that atractylenolide III (the primary bioactive component in ARA) also enhanced PGC1α and AMPK activities in C2C12 cells [16]. However, no reports have been previously issued on the regulation of energy metabolism by ARA in an obese animal model. Here, we investigated the effects of ARA on obesity, glucose intolerance and energy metabolism in skeletal muscle and brown fat in a mouse model of obesity.

## 2. Results

### 2.1. ARA Reduced Body Weight Gain

**Figure 1:**
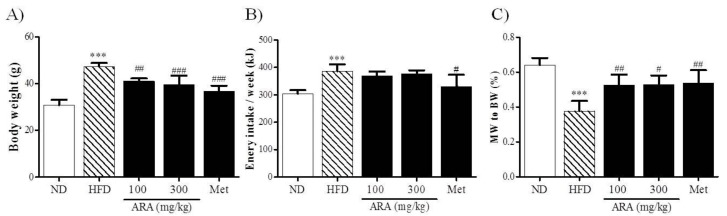
Effects of ARA on body weight. (**A**) body weight; (**B**) energy intake; (**C**) gastrocnemius muscle to body weight ratios. Results are presented as means ± standard errors of the mean (SEM) (*n* = 5). *** *p* < 0.001 versus normal chow fed controls (the ND group); ^###^ *p* < 0.001, ^##^ *p* < 0.01, ^#^ *p* < 0.05 versus the high fat diet fed mice (the HFD group). ARA, Atractylodis Rhizoma Alba; ND, normal diet; HFD, high fat diet; BW, body weight; MW, muscle weight.

After the 16-week experimental period, HFD mice were heavier than normal diet (ND) mice. ARA at 100, and 300 mg/kg and metformin at 250 mg/kg significantly reduced body weight gain (Figure 1A). As was expected, energy intake was higher in the HFD group than in the ND group, but remarkably energy intakes were no higher in the ARA groups versus HFD controls, whereas metformin significantly reduced intake versus HFD controls (Figure 1B). Regarding skeletal muscles, obesity-induced muscle loss was observed in the HFD group, and this reduction was diminished by ARA administration (Figure 1C).

### 2.2. ARA Ameliorated Obesity-Induced Glucose Intolerance

**Figure 2:**
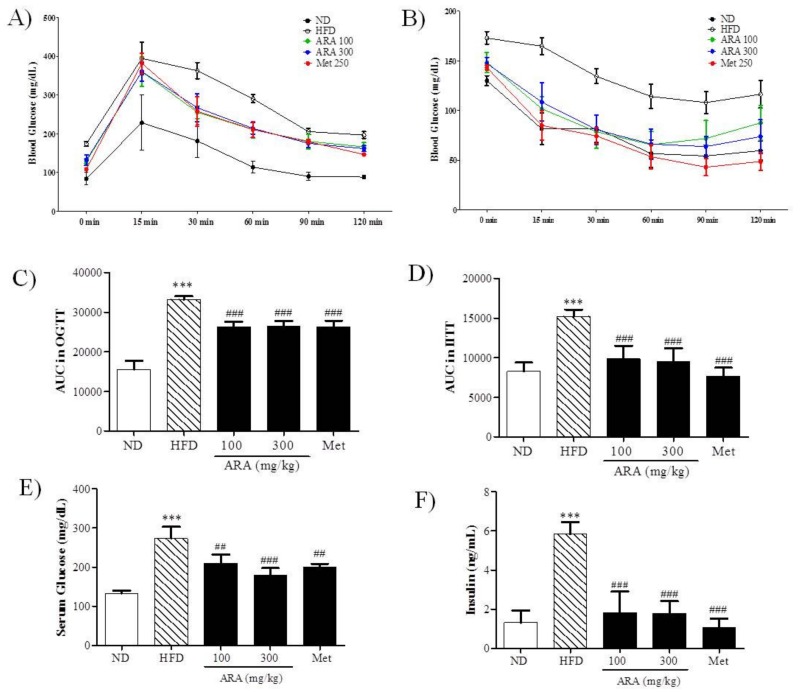
Effects of ARA on glucose metabolism. (**A**) oral glucose tolerance test (OGTT); (**B**) intraperitoneal insulin tolerance test (IITT); (**C**) AUC in OGTT; (**D**) AUC in IITT; (**E**) fasting serum glucose; (**F**) serum insulin. Results are presented as means ± standard errors of the mean (SEM) (*n* = 5). *** *p* < 0.001 versus normal chow fed controls (the ND group); ^###^ *p* < 0.001, ^##^ *p* < 0.01 versus the high fat diet fed mice (the HFD group). AUC, area under the curve.

An oral glucose test (OGTT) and an intraperitoneal insulin tolerance test (IITT) were used to determine the effects of ARA on glucose tolerance and insulin sensitively, respectively (Figure 2A,B). As was expected, glucose tolerance was impaired in the HFD group as compared with ND controls, whereas both ARA groups and the metformin (Met) group had significantly better and similar glucose tolerances as determined by area under the curve (AUC) analysis (Figure 2C). AUC areas of IITT results also showed that ARA treatment significantly decreased insulin resistance versus HFD controls (Figure 2D). Serum glucose and insulin levels were notably decreased by ARA treatment, and these reductions were similar to those observed in the Met group (Figure 2E,F).

### 2.3. ARA Reduced Obesity-Induced Lipid Accumulation and Improved Serum Parameters

**Figure 3:**
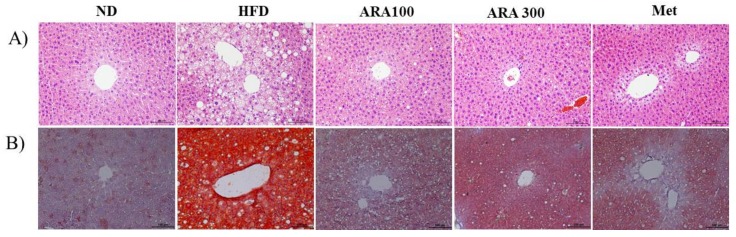
Effects of ARA on lipid accumulations in livers. (**A**) hematoxylin and eosin staining in liver tissues; (**B**) oil red O stained in liver tissues, original magnification 200×. Scale bar: 100 µm.

Mice in the HFD group showed intense lipid accumulation in liver as determined by hematoxylin and eosin (H&E) and oil-red-O staining. ARA and metformin both decreased lipid accumulation (Figure 3A,B). Liver function enzymes, serum alanine aminotransferase (ALT) and aspartate aminotransferase (AST), were significantly increased by HFD group versus ND controls, but ARA treatment decreased HFD-induced serum levels. Serum total cholesterol (TC) levels were also significantly increased by HFD, but ARA treatment at both doses significantly reduced these increases. Serum alkaline phosphatase (ALP), triglycerides (TG) and high-density lipoprotein cholesterol (HDL-C) levels were unaffected by the HFD (Table 1).

### 2.4. ARA Regulated Energy Metabolism in Skeletal Muscle

**Figure 4:**
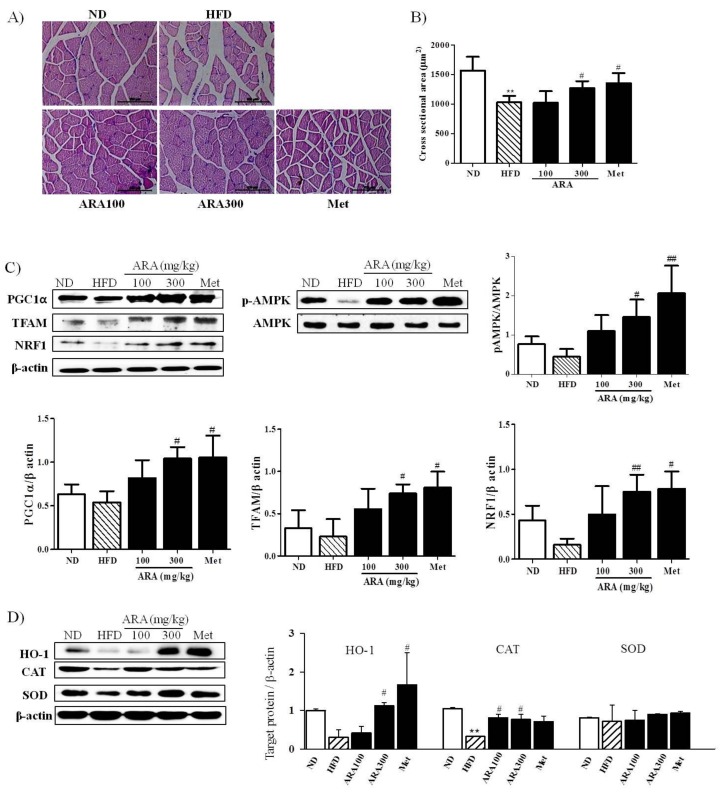
Effects of ARA on energy metabolism in skeletal muscle. (**A**) muscle fiber morphologies of gastrocnemius muscle, original magnification 400×, Scale bar: 50 µm.; (**B**) cross-sectional area of muscle fibers; (**C**) PGC1α, TFAM, NRF1 and pAMPK protein levels in skeletal muscle tissues; (**D**) HO-1, SOD and CAT protein levels in skeletal muscle tissues. Results are presented as means ± standard errors of the mean (SEM) (*n* = 3). ** *p* < 0.01 versus normal chow fed controls (the ND group); ^##^ *p* < 0.01, ^#^ *p* < 0.05 versus the high fat diet fed mice (the HFD group). PGC1α, peroxisome proliferator-activated receptor gamma coactivator 1 alpha; TFAM, mitochondrial transcription factor; NRF1, nuclear respiratory factor 1; AMPK, adenosine monophosphate-activated protein kinase; HO-1, Heme oxygenase-1; SOD, superoxide dismutase; CAT, Catalase.

Obesity accelerates skeletal muscle loss and this loss is strongly associated with obesity-induced insulin resistance [17]. We found that ARA prevented skeletal muscle loss as shown in Figure 1C. Skeletal muscle fiber sizes as determined by histological examinations of gastrocnemius cross sections showed that diameters were greater in the ARA group (300 mg/kg) than in the HFD group (Figure 4A,B).

To understand the molecular basis responsible for lipid accumulation suppressions, glucose tolerance improvements and muscle atrophy inhibition by ARA, we assessed the expressions of PGC1α and AMPK in skeletal muscle. As shown in Figure 4C, ARA (300 mg/kg) increased PGC1α and phosphorylated AMPK protein levels, whereas both were reduced by the HFD. PGC1α is a co-activator of nuclear respiratory factor 1 (NRF1) and mitochondrial transcription factor (TFAM), which are important for mitochondrial biogenesis [18], ARA at 300 mg/kg was found to increase the expressions of NRF1 and TFAM. In addition, because PGC1α is known to regulate oxidative stress induced by obesity [19], we also investigated antioxidative enzyme activities. ARA treatment was found to increase the protein expression of heme oxygenase-1 (HO-1) and catalase as compared with HFD controls (Figure 4D).

### 2.5. ARA Increased Energy Metabolism in Brown Adipose Tissue

**Figure 5:**
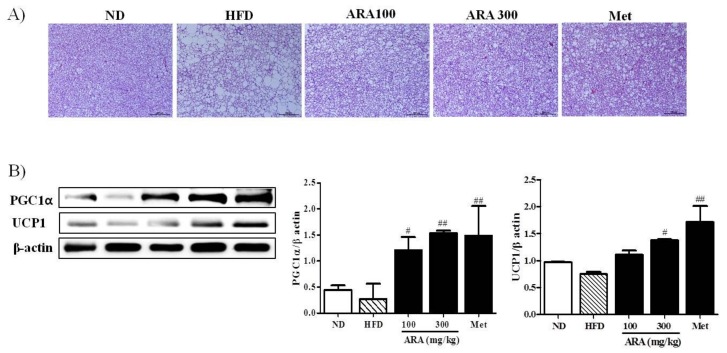
Effects of ARA on energy metabolism in brown fat. (**A**) hematoxylin and eosin staining in brown adipose tissues, original magnification 100×, Scale bar: 200 µm; (**B**) PGC1α and UCP1 protein levels in brown adipose tissues. Results are presented as means ± standard errors of the mean (SEM) (*n* = 3). ^##^ *p* < 0.01, ^#^ *p* < 0.05 versus the high fat diet fed mice (the HFD group). UCP1, uncoupling protein.

Brown fat is also critical for thermogenesis in mice [4], and thus we examined the effects of ARA on brown fat morphology and on the gene expressions of PGC1α and uncoupling protein 1 (UCP1). As compared with HFD mice, brown adipocyte differentiation was enhanced in ARA treated groups, suggesting higher thermogenic activity (Figure 5A). To understand the molecular basis of this observation, we investigated the expression of two thermogenesis related genes, PGC1α and UCP1, in brown fat [20]. We found that the protein levels of both were significantly elevated in the ARA groups as compared with HFD controls (Figure 5B).

## 3. Discussion

Obesity is a global epidemic and an increasing threat to public health [21]. During recent decades, several anti-obesity drugs have been withdrawn from the market because of reported adverse effects [22], and, after years of no new drug introduction, the US Food and Drug Administration (FDA) recently approved multiple new anti-obesity drugs. However, these drugs and previously approved drugs control energy intake by suppressing appetite or fat absorption, and thus no currently available approved drug targets energy expenditure [23], which is believed to provide a more effective, safer means of weight reduction [2]. ARA has long been used in Eastern Asia, including Korea and China, to treat obesity and type 2 diabetes mellitus but the mechanism responsible for its anti-obesity effects has not been elucidated. Here, we report ARA improves obesity and glucose metabolism in obese mice and suggest that this effect is connected with increased energy metabolism in skeletal muscle and brown adipose tissue (BAT).

C5BL/6 mice are susceptible to diet-induced obesity and diabetes [24], and, as was expected, feeding these mice for 16 weeks with an HFD induced significant body weight gain and glucose intolerance. In the present study, we administrated ARA at 100 or 300 mg/kg daily, as has been performed in previous studies [13,14]. ARA was found to dose-dependently reduce weight gain and improve lipid profiles without significantly changing energy intake, which is consistent with the findings of previous studies [13,14]. On the other hand, metformin reduced body weight gain with an decrease of energy intake, which is consistent with our previous observations [25]. Several reports indicate that the anorexic effect of metformin is probably due to the central regulation of appetite [26,27,28]. Mouse models of HFD-induced obesity have been reported to exhibit liver steatosis due to the hepatic accumulations of triglycerides in hepatocytes [29]. In the present study, ARA reduced lipid accumulation in liver tissues and improved liver enzyme functions. Furthermore, diet-induced obesity leads to insulin resistance accompanied by impaired glucose tolerance and insulin sensitivity, and we also found that HFD fed mice exhibited higher fasting glucose, insulin levels and glucose intolerance. ARA treatment decreased these HFD-induced indexes of glucose metabolism to an extent similar to metformin. Accordingly, our observations indicate that ARA improved obesity and glucose metabolism in HFD-induced obese mice but did not affect energy intake, which suggests that dietary ARA might increase in energy metabolism.

To investigate the effect of ARA on energy metabolism at the molecular level, we assessed PGC1α expression in skeletal muscle and BAT. PGC1α is a member of a family of transcription co-activators [30], and is involved in a variety of biological responses, such as mitochondrial biogenesis, glucose/fatty acid metabolism, fiber type switching, and antioxidant defense [19,31]. AMPK is another enzyme that plays a central role in cellular energy metabolism, and supports ATP generation, glucose uptake, glycolysis, fatty acid oxidation, and mitochondrial biogenesis [32], and also has been reported to affect PGC1α activity directly by phosphorylation [33]. Interestingly, in the present study, ARA increased PGC1α and phosphorylated AMPK protein levels and the protein levels of NRF1 and TFAM, which are both downstream transcription factors of PGC1α [18]. Furthermore, these findings are consistent with those that we previously reported for C2C12 skeletal muscle cells [15,16]. In addition, PGC1α has been shown to regulate the expressions of endogenous antioxidant proteins [19], and, in the present study, we observed that ARA also increased levels of antioxidant enzymes, such as HO-1 and catalase. Skeletal muscle morphologies were examined because skeletal muscle is the main determinant of energy expenditure [34], and obesity has been reported to induce skeletal muscle loss [17], and the preservation of muscle mass has been reported to help maintain energy expenditure [34]. Recently, it was reported that, importantly, PGC1α mediates muscle mass [35] and increases oxidative fiber differentiation [35]. In the present study, ARA prevented skeletal muscle loss induced by HFD, as indicated by skeletal muscle mass and fiber diameters. Taken together, it appears likely that the induction of PGC1α activity may underlie the mechanism by which ARA stimulates glucose and fatty acid metabolism and prevents skeletal muscle loss. However, to define an effect of ARA on skeletal muscle mass and fiber type, further investigation should be made at a molecular level, such as myosin heavy chains.

Brown fat is a specialized type of fat that can increase energy expenditure and produce heat. Thus, increasing brown fat amount and/or increasing its activity can increase energy expenditure, and thus have a negative effect on obesity. Brown fat can also improve glucose metabolism and blood lipid levels independent of weight loss [20]. In the present study, ARA increased brown fat amounts and activities, suggesting that it might improve glucose intolerance and lower serum total cholesterol levels. PGC1α is a master regulator of mitochondrial biogenesis and oxidative metabolism, and, in brown fat, can induce the expression of UCP1 and other thermogenic components [4,20]. In the present study, ARA increased brown fat amounts and activities, as made evident by an increase in the protein expressions of PGC1α and UCP1. Accordingly, our findings suggest that the metabolic activities of ARA may be related to PGC1α activation in brown fat and skeletal muscle at a molecular level. To clarify the effect of ARA on energy metabolism, further studies are required to measure its effects on energy expenditure and oxygen consumption.

The present study shows that ARA reduces HFD-induced obesity and glucose intolerance in mouse models of obesity, and indicates that the mechanism responsible for these effects is related to the promotion of energy metabolism in skeletal muscle and brown adipose tissue. We speculate that ARA offers a potential means for developing novel therapeutics for the treatment of obesity and metabolic syndrome.

## 4. Materials and Methods

### 4.1. Preparation of ARA Extract

The dried roots of ARA were purchased from Kwangmyungdang Medicinal Herbs (Ulsan, Korea), ground, and boiled in purified water for 3 h. The aqueous extracts were filtered through two layers of Whatman No. 3 filter paper and concentrated under vacuum evaporation (Rotavapor R-144 system, Buchi, Switzerland). The extracts were dried with a freeze dryer (FD5508A, Ilsin Lab, Dongducheon, Korea), the yield was 26.0% and stored at −80 °C. In our previous study, we detected atractylenolide III using HPLC analysis [15], and we also separated atractylodin in the present study (Figure S1).

### 4.2. Animals and Experimental Design

All experimental procedures were approved beforehand by the Animal Experimentation Ethical Committee at Dongguk University (Permit number: 2016-0624). C5BL/6 mice (aged 5 weeks, male) were purchased from Samtako Bio Korea (Osan, Geonggido, Korea) and kept in individual cages at 22–23 °C under a 12 h-light/12 h-dark cycle. Animals were acclimatized for a week and provided a normal chow diet with ad libitum access to water. They were then randomly allocated to 5 groups of 5 mice each as follows; (1) an ND group: fed 18 kcal% fat diet (2018S, Envigo, Huntingdon, CBE, UK), (2) an HFD group: fed 60 kcal% fat diet (D12492, Research Diets, New Brunswick, NJ, USA), (3) an HFD plus ARA 100 mg/kg/day group (ARA 100), (4) an HFD plus ARA 300 mg/kg/day group (ARA 300), and (5) an HFD plus metformin 250 mg/kg/day group (Met) (positive control) for 16 weeks. Body weights were similar in the 5 groups at baseline. ARA and Met were administered orally by gavage, and animals in the ND and HFD groups were given an equal volume of distilled water by gavage. Body weights, food consumptions, and blood glucose levels were measured weekly. After the treatment period, all mice were fasted for 12 h and sacrificed. Livers, brown adipose tissues, pancreases and gastrocnemius muscle tissues were collected and weighed.

### 4.3. Oral Glucose Tolerance Testing

OGTT (2 g/body weight, kg) was carried out after the 16-week feeding and gavage period. All mice were fasted overnight before testing. Blood samples were taken from a tail vein at 0, 15, 30, 60, 90, and 120 min after glucose administration, and blood glucose levels were measured using a glucometer (Roche Diagnostics, Basel, Switzerland).

### 4.4. Intraperitoneal Insulin Tolerance Testing

For IITT, food was removed immediately before testing and animals were administrated human regular insulin (0.5 U/kg body weight, i.p.). Blood samples were collected from a tail vein at 0, 30, 90, and 120 min after injection.

### 4.5. Serum Analysis

Blood samples were collected by cardiac puncture, and serum was obtained by centrifuging at 5000 *g* for 15 min after sacrifice. TG, TC, and HDL cholesterol levels were measured using commercial colorimetric kits (Asan Pharm. Co., Seoul, Korea). Serum hepatic enzyme activities were determined using AST, ALT and ALP kits (Asan Pharm. Co.). Serum insulin levels were analyzed using a mouse insulin ELISA kit (EMD Millipore Corporation, Billerica, MA, USA).

### 4.6. Histology Analysis

Livers, brown adipose, and skeletal muscle tissues were fixed in 10% formalin, stained with hematoxylin and eosin (H&E), and then observed under a Leica DM 2500 microscope (Leica, Wetzlar, Germany). Cross-sectional areas of adipose and skeletal muscle tissues were quantified using ImageJ analysis software (version 1.43, NIH, Bethesda, MD, USA) [36].

### 4.7. Lipid Accumulation Analysis in Liver

To observe lipid accumulation in liver, frozen tissues were stained with oil red O. Optimal cutting temperature-embedded liver tissues were sectioned at 10 μm and mounted on clear cryostat slides. Tissues were allowed to dry for 1 h, hydrated in distilled water for 5 min, dipped in 100% propylene glycol for 2 min, stained with oil red O working solution (Sigma-Aldrich, St. Louis, MO, USA) for 1 h, dipped in 85% propylene glycol solution for 1 min, and rinsed with distilled water. Sections were visualized under a Leica DM 2500 microscope.

### 4.8. Western Blot

Muscles and brown fat were collected and frozen immediately. Whole-cell lysates were prepared in a lysis buffer by sonications as described previously [25]. The antibodies used and their sources were as follows: anti-phospho-AMPKα (Thr 172), anti-AMPKα (Cell Signaling Technology, Danvers, MA, USA), anti-PGC1α, anti-NRF1, anti-TFAM, anti-UCP1, anti-HO-1, anti-superoxide dismutase (SOD), anti-CAT, and anti-β-actin (Sigma-Aldrich, St. Louis, MO, USA). Bands were developed using Western detection reagent (GE Healthcare Bio-Sciences, Pittsburgh, PA, USA) and quantified by densitometry using ImageJ.

### 4.9. Statistical Analysis

The analysis was conducted by one-way ANOVA followed by Tukey’s post hoc test in GraphPad Prism program ver 5.0 (GraphPad Software, La Jolla, CA, USA). Results are presented as means ± standard errors of means (SEMs). Statistical significance was accepted for *p*-values < 0.05.

## Figures and Tables

**Table 1:** Effect of Atractylodis Rhizoma Alba (ARA) on serum profiles.

Serum	ND	HFD	ARA 100	ARA 300	Met
ALT (IU/L)	8.96 ± 4.95	54.84 ± 16.73 ***	12.88 ± 7.76 ^###^	10.53 ± 4.74 ^###^	11.95 ± 1.88 ^###^
AST (IU/L)	44.06 ± 1.40	58.03 ± 7.45 *	47.16 ± 4.72 *	49.02 ± 7.88	61.76 ± 12.87
ALP (IU/L)	8.42 ± 0.25	8.63 ± 0.86	7.54 ± 0.45	7.32 ± 0.77	7.99 ± 0.62
TG (mg/dL)	42.75 ± 0.63	42.95 ± 0.39	41.75 ± 0.76	40.38 ± 0.88 ^###^	40.70 ± 0.31 ^###^
TC (mg/dL)	225.86 ± 9.84	276.61 ± 27.01 *	223.59 ± 11.09 ^##^	204.46 ± 26.11 ^###^	182.55 ± 7.85 ^###^
HDL-C (mg/dL)	73.26 ± 8.25	107.52 ± 12.68 **	82.87 ± 5.31	94.48 ± 18.75	69.96 ± 5.36 ^###^

Results are presented as means ± standard errors of the mean (SEM) (*n* = 5). *** *p* < 0.001, ** *p* < 0.01, * *p* < 0.05 versus normal chow fed controls (the ND group); ^###^ *p* < 0.001, ^##^ *p* < 0.01 versus the high fat diet fed mice (the HFD group). ALT, alanine aminotransferase; AST, aspartate aminotransferase; ALP, alkaline phosphatase; TG, triglycerides; TC, total cholesterol; and HDL-C, high-density lipoprotein cholesterol.

## Supplementary Materials

The following are available online at www.mdpi.com/1422-0067/19/1/278/s1.

## Abbreviations

ALP	Alkaline phosphatase
ALT	Alanine aminotransferase
AMPK	Adenosine monophosphate-activated protein kinase
ARA	Atractylodis rhizoma alba
AST	Aspartate aminotransferase
AUC	Area under the curve
BAT	Brown adipose tissue
BW	Body weight
CAT	Catalase
H&E	Hematoxylin and eosin
HDL-C	High-density lipoprotein cholesterol
HFD	High-fat diet
HO-1	Heme oxygenase-1
IITT	Intraperitoneal insulin tolerance test
Met	Metformin
MW	Muscle weight
ND	Normal diet
NRF1	Nuclear respiratory factor 1
OGTT	Oral glucose tolerance test
PGC1α	Peroxisome proliferator-activated receptor gamma coactivator 1 alpha
SOD	Superoxide dismutase
TC	Total cholesterol
TCM	Traditional Chinese medicine
TFAM	Mitochondrial transcription factor
TG	Triglycerides
TKM	Traditional Korean medicine
UCP1	Uncoupling protein 1

## References

[1] Moller D.E. (2001). New drug targets for type 2 diabetes and the metabolic syndrome. Nature.

[2] Tseng Y.-H., Cypess A.M., Kahn C.R. (2010). Cellular bioenergetics as a target for obesity therapy. Nat. Rev. Drug Discov..

[3] Duchamp C., Barre H. (1993). Skeletal muscle as the major site of nonshivering thermogenesis in cold-acclimated ducklings. Am. J. Physiol..

[4] Cypess A.M., Kahn C.R. (2010). Brown fat as a therapy for obesity and diabetes. Curr. Opin. Endocrinol. Diabetes Obes..

[5] Krssak M., Petersen K.F., Dresner A., DiPietro L., Vogel S., Rothman D., Shulman G., Roden M. (1999). Intramyocellular lipid concentrations are correlated with insulin sensitivity in humans: A ^1^H NMR spectroscopy study. Diabetologia.

[6] Li X.-T., Kuang H.-X., Zhao J. (2015). Why Is Qi-Invigorating Therapy in Chinese Medicine Suitable for Mitochondrial Diseases? A Bioenergetic Perspective.

[7] Wallace D.C. (2008). Mitochondria as chi. Genetics.

[8] Kim S.J., Shin S.W., Kim H.J. (2003). Obesity from the viewpoint of metabolic rate. J. Korean Med. Obes. Res..

[9] Li X.-T. (2012). Investigation on the Mechanism of Qi-Invigoration from a Perspective of Effects of Sijunzi Decoction on Mitochondrial Energy Metabolism.

[10] Lee S.H., Lee H.J., Lee Y.H., Lee B.W., Cha B.S., Kang E.S., Ahn C.W., Park J.S., Kim H.J., Lee E.Y. (2012). Korean red ginseng (*Panax ginseng*) improves insulin sensitivity in high fat fed Sprague-Dawley rats. Phytother. Res..

[11] The Korea Association of Herbology (1991). Herbology.

[12] Huang Y., Wang L., Wang S., Cai F., Zheng G., Lu A., Yu X., Jiang M. (2013). Treatment principles of obesity with chinese herbal medicine: Literature analysis by text mining. Engineering.

[13] Wang J.-H., Bose S., Kim H.-G., Han K.-S., Kim H. (2015). Fermented rhizoma *Atractylodis macrocephalae* alleviates high fat diet-induced obesity in association with regulation of intestinal permeability and microbiota in rats. Sci. Rep..

[14] Kim C.K., Kim M., Oh S.D., Lee S.-M., Sun B., Choi G.S., Kim S.-K., Bae H., Kang C., Min B.-I. (2011). Effects of *Atractylodes macrocephala Koidzumi* rhizome on 3T3-L1 adipogenesis and an animal model of obesity. J. Ethnopharmacol..

[15] Song M.Y., Kang S.Y., Oh T.W., Kumar R.V., Jung H.W., Park Y.-K. (2015). The roots of *Atractylodes macrocephala Koidzumi* enhanced glucose and lipid metabolism in C2C12 myotubes via mitochondrial regulation. Evid. Based Complement Alternat. Med..

[16] Song M.Y., Jung H.W., Kang S.Y., Park Y.-K. (2017). Atractylenolide III enhances energy metabolism by increasing the SIRT-1 and PGC1α expression with AMPK phosphorylation in C2C12 mouse skeletal muscle cells. Biol. Pharm. Bull..

[17] Kalyani R.R., Corriere M., Ferrucci L. (2014). Age-related and disease-related muscle loss: The effect of diabetes, obesity, and other diseases. Lancet Diabetes Endocrinol..

[18] Jornayvaz F.R., Shulman G.I. (2010). Regulation of mitochondrial biogenesis. Essays Biochem..

[19] Kang C., Li Ji L. (2012). Role of PGC-1α signaling in skeletal muscle health and disease. Ann. N. Y. Acad. Sci..

[20] Kim S.H., Plutzky J. (2016). Brown fat and browning for the treatment of obesity and related metabolic disorders. Diabetes Metab. J..

[21] Caballero B. (2007). The global epidemic of obesity: An overview. Epidemiol. Rev..

[22] Onakpoya I.J., Heneghan C.J., Aronson J.K. (2016). Post-marketing withdrawal of anti-obesity medicinal products because of adverse drug reactions: A systematic review. BMC Med..

[23] Daneschvar H.L., Aronson M.D., Smetana G.W. (2016). FDA-approved anti-obesity drugs in the United States. Am. J. Med..

[24] Wang C.-Y., Liao J.K. (2012). A mouse model of diet-induced obesity and insulin resistance. Methods Mol. Biol..

[25] Jung H.W., Kang A.N., Kang S.Y., Park Y.-K., Song M.Y. (2017). The root extract of *Pueraria lobata* and its main compound, puerarin, prevent obesity by increasing the energy metabolism in skeletal muscle. Nutrients.

[26] Rouru J., Huupponen R., Pesonen U., Koulu M. (1992). Subchronic treatment with metformin produces anorectic effect and reduces hyperinsulinemia in genetically obese Zucker rats. Life Sci..

[27] Rouru J., Pesonen U., Koulu M., Huupponen R., Santti E., Virtanen K., Rouvari T., Jhanwar-Uniyal M. (1995). Anorectic effect of metformin in obese Zucker rats: Lack of evidence for the involvement of neuropeptide Y. Eur. J. Pharmacol..

[28] Lv W.-S., Wen J.-P., Li L., Sun R.-X., Wang J., Xian Y.-X., Cao C.-X., Wang Y.-L., Gao Y.-Y. (2012). The effect of metformin on food intake and its potential role in hypothalamic regulation in obese diabetic rats. Brain Res..

[29] Xu J., Lloyd D.J., Hale C., Stanislaus S., Chen M., Sivits G., Vonderfecht S., Hecht R., Li Y.-S., Lindberg R.A. (2009). Fibroblast growth factor 21 reverses hepatic steatosis, increases energy expenditure, and improves insulin sensitivity in diet-induced obese mice. Diabetes.

[30] Liang H., Ward W.F. (2006). PGC-1α: A key regulator of energy metabolism. Adv. Physiol. Educ..

[31] Liu C., Lin J.D. (2011). PGC-1 coactivators in the control of energy metabolism. Acta Biochim. Biophys. Sin..

[32] O’Neill H.M., Holloway G.P., Steinberg G.R. (2013). AMPK regulation of fatty acid metabolism and mitochondrial biogenesis: Implications for obesity. Mol. Cell. Endocrinol..

[33] Jäger S., Handschin C., St-Pierre J., Spiegelman B.M. (2007). AMP-activated protein kinase (AMPK) action in skeletal muscle via direct phosphorylation of Pgc-1α. Proc. Natl. Acad. Sci. USA.

[34] Zurlo F., Larson K., Bogardus C., Ravussin E. (1990). Skeletal muscle metabolism is a major determinant of resting energy expenditure. J. Clin. Investig..

[35] Sandri M., Lin J., Handschin C., Yang W., Arany Z.P., Lecker S.H., Goldberg A.L., Spiegelman B.M. (2006). Pgc-1α protects skeletal muscle from atrophy by suppressing foxo3 action and atrophy-specific gene transcription. Proc. Natl. Acad. Sci. USA.

[36] Parlee S.D., Lentz S.I., Mori H., MacDougald O.A. (2014). Quantifying size and number of adipocytes in adipose tissue. Methods Enzymol..

