# Supplementary material for: The Root of Atractylodes macrocephala Koidzumi Prevents Obesity and Glucose Intolerance and Increases Energy Metabolism in Mice
Source: Int J Mol Sci. 2018 Jan 17;19(1):278. doi: 10.3390/ijms19010278 (PMC5796224; doi:10.3390/ijms19010278)
Supplement: Supplementary file 1 [file ijms-19-00278-s001.pdf]

Supplementary material

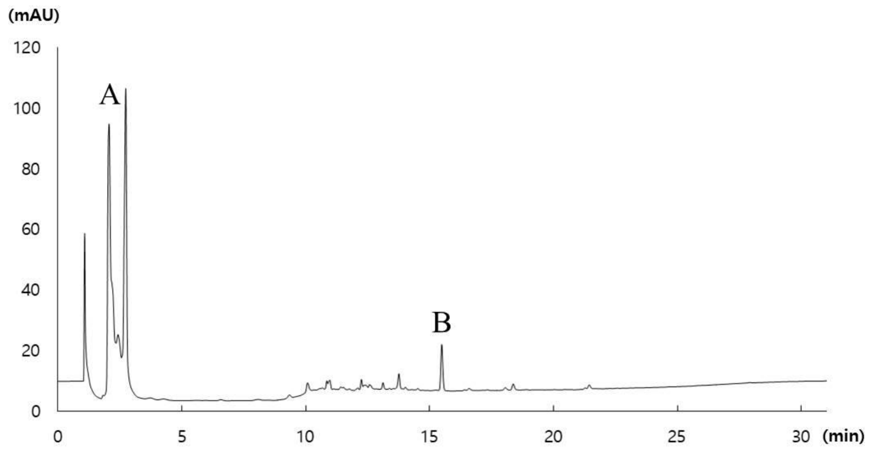

Figure S1. HPLC fingerprint of ARA. (A) atractylodin; (B) atractylenolide III. HPLC, High Performance Liquid Chromat; ARA, Atractylodis Rhizoma Alba.

Supplementaly Materials and Methods

HPLC anaylsis

HPLC was performed using an Agilent 1220 Infinity LC System (Agilent Technologies, Santa Clara ,CA, USA) and an Agilent Eclipse XDB-C18 column (4.6 mm x250 mm, 5 μm) (Agilent Technologies). HPLC quality water and methanol were used as a mobile phase with gradient elution at a flow rate of 0.5 mL/min. 20 μL of ARA extract (50 mg/mL), atractylodin (500 μM) (the isolated standard compound [1] was supplied by professor Chin.) and atractylenolide III (100 μM) (the standard compound, Sigma-Aldrich, St. Louis, MO, USA) injected and detected at 220 nm using a DAD detector.

1. Chae, H.S.; Kim, Y.M.; Chin, Y.W. Atractylodin inhibits interleukin-6 by blocking NPM-ALK activa tion and MAPKs in HMC-1. Molecules 2016,21,1169, doi: 10.3390/molecules21091169. Available onli ne: https://www.ncbi.nlm.nih.gov/pubmed/27598116.
